# Cognitive processing of semantic mismatch in Chinese reading: the moderating effect of contextual constraint

**DOI:** 10.3389/fpsyg.2026.1802733

**Published:** 2026-06-18

**Authors:** Yunfei Liu, Biqing Zou, Jing Yang

**Affiliations:** School of Foreign Studies, China Three Gorges University, Yichang, Hubei, China

**Keywords:** Chinese reading, contextual constraint, eye movements, predictive processing, semantic mismatch

## Abstract

**Background:**

Semantic mismatch processing is fundamental to language comprehension, yet how contextual constraint modulates this processing in Chinese reading remains unclear. The present study investigated the cognitive processing of semantic mismatch in Chinese modifier-noun constructions and examined the moderating effect of contextual constraint.

**Methods:**

Forty-eight native Mandarin Chinese speakers read sentences containing semantically matched or mismatched modifier-noun constructions embedded in high- or low-constraint contexts while their eye movements were recorded using an EyeLink 1000 Plus eye tracker. Linear mixed-effects models were used to analyze eye movement measures.

**Results:**

Robust semantic mismatch effects were observed: mismatched constructions elicited longer first fixation durations and gaze durations, higher regression probabilities, and longer total reading times. Critically, significant interactions between semantic relation and contextual constraint emerged on gaze duration and late processing measures, with mismatch effects approximately twice as large in high-constraint contexts. Simple effects analyses revealed that contextual constraint selectively affected mismatched constructions while leaving matched constructions unaffected.

**Conclusion:**

These findings support predictive processing accounts of language comprehension, demonstrating that readers actively generate contextual expectations and experience amplified processing difficulty when these expectations are violated.

## 1 Introduction

Language comprehension requires readers to rapidly integrate multiple sources of information to construct coherent mental representations. Semantic processing lies at the heart of this process, and when semantic information conflicts or fails to integrate smoothly, readers experience processing difficulty. Understanding how readers detect and process semantic anomalies provides valuable insights into the fundamental mechanisms of language comprehension (Kutas and Federmeier, 2011; Kuperberg et al., 2020).

Semantic mismatch occurs when the meaning of one linguistic element conflicts with another element with which it must be combined. At the phrasal level, modifier-noun constructions represent a particularly interesting case for studying semantic integration. In these constructions, the modifier must be semantically compatible with the head noun. When a modifier violates the selectional restrictions of its head noun, as in expressions like “boiling hot ice cubes,” readers encounter a local semantic anomaly that disrupts comprehension processes (Warren et al., 2015; Haeuser and Kray, 2022).

The modifier-noun construction, known as the definite-central structure (定中结构) in Chinese, is one of the most fundamental syntactic patterns in Mandarin Chinese. This construction typically consists of an attributive modifier followed by a head noun. When the semantic relationship between modifier and head noun is violated, readers must detect this incongruity and attempt to resolve it. The cognitive processes underlying this detection and resolution are not yet fully understood, particularly in Chinese reading (Li and Pollatsek, 2020; Liu and Ni, 2024).

Contextual information plays a crucial role in shaping how readers process linguistic input. The preceding context can establish expectations about upcoming content, and these expectations can facilitate processing when confirmed or disrupt processing when violated. High-constraint contexts strongly predict particular continuations, while low-constraint contexts leave many possibilities open. When a high-constraint context has led readers to expect a particular phrase, encountering a semantically anomalous expression may produce particularly severe disruption, as readers must not only detect the local incongruity but also revise their contextually generated expectations (Szewczyk and Federmeier, 2022; Huizeling et al., 2022).

Predictive processing has emerged as a central framework for understanding context effects in language comprehension. According to this framework, readers actively generate predictions about upcoming content and pre-activate linguistic representations accordingly. When predictions are violated, the degree of disruption depends on prediction strength. This framework predicts that semantic mismatch effects should be larger in high-constraint contexts, where strong predictions are violated, than in low-constraint contexts (Ryskin and Nieuwland, 2023; Stone et al., 2023; Gastaldon et al., 2024).

Despite the theoretical appeal of predictive processing accounts, the behavioral consequences of semantic anomaly specifically within constraining contexts have only recently received direct empirical attention. Ness and Meltzer-Asscher (2018) demonstrated that encountering a word that violates a strong contextual prediction carries measurable processing costs beyond those attributable to local semantic incongruity alone, with failed predictions producing lexical inhibition effects at both the behavioral and electrophysiological levels. Converging eye-tracking evidence comes from Wong et al. (2024), who found that processing costs in strongly constraining contexts were amplified specifically for anomalous words but not for plausible yet unpredictable continuations, suggesting that the interaction between contextual constraint and semantic anomaly reflects a qualitatively distinct processing challenge. However, this behavioral evidence derives predominantly from studies examining single-word targets in alphabetic languages, leaving open the question of whether analogous modulation operates at the phrasal level and across typologically distinct writing systems.

Chinese presents a unique testing ground for these theories. The absence of explicit word boundaries in Chinese means that lexical segmentation cannot rely on orthographic cues alone but must instead be guided substantially by contextual and semantic information processed on-line. This dependence on context for basic word identification may render Chinese readers particularly sensitive to contextual expectations, potentially amplifying the consequences of constraint violations relative to alphabetic language readers for whom orthographic word boundaries provide an independent segmentation scaffold. Furthermore, the modifier-noun construction is one of the most productive and frequently encountered syntactic patterns in Mandarin, meaning that semantic integration within this construction represents a routine and well-practiced component of Chinese reading comprehension rather than an incidental processing event. These structural characteristics make Chinese reading a theoretically motivated testing ground for examining whether the interaction between semantic anomaly and contextual constraint documented in alphabetic languages reflects a universal property of the human language comprehension system or whether it is modulated by writing system-specific factors (Li et al., 2022; Zhang et al., 2022).

Eye-tracking methodology provides an ideal tool for investigating reading processes. Different eye movement measures reflect different processing stages: early measures such as first fixation duration and gaze duration reflect initial word recognition, while late measures such as total reading time and regression probability reflect later integration and re-analysis processes (Rayner, 1998; Pan et al., 2022; Xia et al., 2023).

The present study aimed to investigate semantic mismatch processing in Chinese modifier-noun constructions and examine how contextual constraint modulates this processing. We predicted that mismatched constructions would elicit longer fixation times and higher regression probabilities than matched constructions. Based on predictive processing accounts, we hypothesized that mismatch effects would be larger in high-constraint contexts than in low-constraint contexts (Brothers et al., 2020; Michaelov et al., 2024).

## 2 Methods

### 2.1 Participants

A total of 48 native Mandarin Chinese speakers (32 females, mean age = 21.8 years, SD = 2.3, range: 18–27 years) were recruited from China Three Gorges University to participate in this study. The sample size was determined by an *a priori* power analysis conducted using GPower 3.1. Based on a medium effect size (*f* = 0.25), an alpha level of 0.05, and a desired power of 0.80 for a 2 × 2 within-subjects design, the minimum required sample size was 36 participants. We recruited additional participants (*N* = 48) to account for potential data loss and to increase statistical power. We acknowledge that simulation-based power analyses using packages such as SIMR (Green and MacLeod, 2016) would more accurately reflect the power of the linear mixed-effects models used in the main analyses. However, as the GPower analysis was conducted prior to data collection and the final sample size of 48 exceeded the minimum requirement by a considerable margin, we are confident that the study was adequately powered to detect effects of the magnitude observed.

All participants had normal or corrected-to-normal vision and reported no history of reading disabilities or neurological disorders. None of the participants had prior exposure to the experimental materials. Each participant provided written informed consent before the experiment and received monetary compensation (40 RMB) or course credit for their participation. This study was approved by the Ethics Committee of China Three Gorges University (Approval No.: CTGU-EC-2025-129).

### 2.2 Design

The experiment employed a 2 (semantic relation: match vs. mismatch) × 2 (contextual constraint: high vs. low) within-subjects design. The dependent variables were eye movement measures, including both early processing measures (skipping rate, first fixation duration, and gaze duration) and late processing measures (regression probability, go-past time, and total reading time).

Experimental sentences were distributed across four lists using a Latin square design to ensure that each participant saw only one version of each sentence while being exposed to all experimental conditions. Each list contained 80 experimental sentences (20 sentences per condition) and 40 filler sentences.

### 2.3 Materials

A total of 80 sets of experimental sentences were constructed, with each set comprising four versions corresponding to the four experimental conditions. All sentences contained a critical modifier-noun construction (e.g., 形容词 + 中心名词 [adjective + head noun]) embedded in sentence contexts that varied in constraint level.

Manipulation of semantic relation. Semantic match and mismatch conditions were created by manipulating the semantic congruency between the modifier and the head noun in the modifier-noun construction. In the match condition, the modifier was semantically compatible with the head noun (e.g., 滚烫的开水 “boiling hot water”). In the mismatch condition, the modifier violated the selectional restrictions of the head noun, creating a semantic anomaly (e.g., 滚烫的冰块 “boiling hot ice cube”; Fritz and Baggio, 2020).

Manipulation of contextual constraint. Contextual constraint was manipulated through the sentence context preceding the target modifier-noun construction. High-constraint contexts strongly predicted the upcoming target construction, whereas low-constraint contexts provided minimal predictive information. The constraint level was determined by a cloze probability task, in which 30 participants (who did not participate in the main experiment) were asked to complete each sentence fragment with the first word or phrase that came to mind. High-constraint sentences had a mean cloze probability of 0.72 (SD = 0.14), while low-constraint sentences had a mean cloze probability of 0.15 (SD = 0.09). These cloze probabilities reflect completions matching the semantically congruent target construction; the semantically mismatched constructions were never produced by participants in the cloze task. The difference was statistically significant, *t*(79) = 31.26, *p* < 0.001. Examples of the four experimental conditions are presented in Table 1.

**Table 1:** Examples of experimental sentences in four conditions.

Contextual constraint	Semantic relation	Example sentence
High	Match	服务员刚烧好水 把**[滚烫的]₁[开水]₂[倒进了]₃**茶壶里。 (The waiter had just boiled the water and poured the **[boiling hot]₁ [water]₂ [into]₃** the teapot.)
High	Mismatch	服务员刚烧好水 把**[滚烫的]₁[冰块]₂[倒进了]₃**茶壶里。 (The waiter had just boiled the water and poured the **[boiling hot]₁ [ice cubes]₂ [into]₃** the teapot.)
Low	Match	服务员走进厨房 把**[滚烫的]₁[开水]₂[倒进了]₃**茶壶里。 (The waiter walked into the kitchen and poured the **[boiling hot]₁ [water]₂ [into]₃** the teapot.)
Low	Mismatch	服务员走进厨房 把**[滚烫的]₁[冰块]₂[倒进了]₃**茶壶里。 (The waiter walked into the kitchen and poured the **[boiling hot]₁ [ice cubes]₂ [into]₃** the teapot.)

The target modifier-noun constructions are presented in bold. Square brackets denote the three regions of interest: [·]₁ = modifier region; [·]₂ = noun region; [·]₃ = spillover region. The sentence context preceding the target construction constitutes the contextual constraint manipulation.

Material controls. To ensure that any observed effects could be attributed to the experimental manipulations rather than confounding variables, the target modifier-noun constructions were carefully matched across conditions on several lexical properties, including word frequency, stroke number, and word length. These variables were obtained from the SUBTLEX-CH database (Cai and Brysbaert, 2010; available at https://www.ugent.be/pp/experimentele-psychologie/en/research/documents/subtlexch) and the Modern Chinese Frequency Dictionary (Language Teaching and Research Institute of Beijing Language Institute, 1986). As shown in Table 2, no significant differences were found between conditions on any of the control variables (all ps > 0.05).

**Table 2:** Lexical properties of target modifier-noun constructions across conditions.

Variable	Match condition	Mismatch condition	*t*	*p*
Modifier frequency (per million)	45.32 (38.67)	45.32 (38.67)	—	—
Head noun frequency (per million)	52.18 (41.25)	48.76 (39.83)	0.53	0.598
Modifier stroke number	8.65 (2.41)	8.65 (2.41)	—	—
Head noun stroke number	9.12 (2.87)	9.45 (3.02)	0.71	0.481
Construction length (characters)	4.20 (0.56)	4.25 (0.54)	0.57	0.572

Standard deviations are presented in parentheses. The same modifiers were used in match and mismatch conditions, so no comparison was needed for modifier-related variables.

Norming studies: Two norming studies were conducted prior to the main experiment. First, a semantic plausibility rating task was administered to 25 participants to verify the manipulation of semantic relation. Participants rated the plausibility of each modifier-noun construction on a 7-point scale (1 = highly implausible, 7 = highly plausible). Results confirmed that match constructions (M = 5.83, SD = 0.76) were rated as significantly more plausible than mismatch constructions (M = 2.14, SD = 0.89), *t*(158) = 28.65, *p* < 0.001.

Second, a cloze probability task was conducted with 30 participants to verify the manipulation of contextual constraint, as described above.

Filler sentences: An additional 40 filler sentences were included to prevent participants from developing strategies based on the experimental manipulations. Filler sentences varied in length and structure and did not contain any semantic anomalies.

### 2.4 Apparatus

Eye movements were recorded using an EyeLink 1,000 Plus eye tracker (SR Research, Ontario, Canada) with a sampling rate of 1,000 Hz. Although viewing was binocular, only the right eye was tracked. Participants were seated approximately 60 cm from a 24-inch monitor with a screen resolution of 1920 × 1,080 pixels. At this viewing distance, approximately 2.8 characters subtended one degree of visual angle. Sentences were displayed in SimSun font with a font size of 22 points in black (RGB: 0, 0, 0) on a white background (RGB: 255, 255, 255). A chin rest and forehead rest were used to minimize head movements during the experiment.

### 2.5 Procedure

Participants were tested individually in a quiet, dimly lit room. After providing informed consent, participants received both written and verbal instructions explaining the task. They were instructed to read each sentence silently at their natural pace for comprehension.

The experiment began with a 9-point calibration and validation procedure. Calibration was accepted when the average error was less than 0.5° and the maximum error was less than 1.0°. Recalibration was performed whenever necessary throughout the experiment.

**Figure 1:**
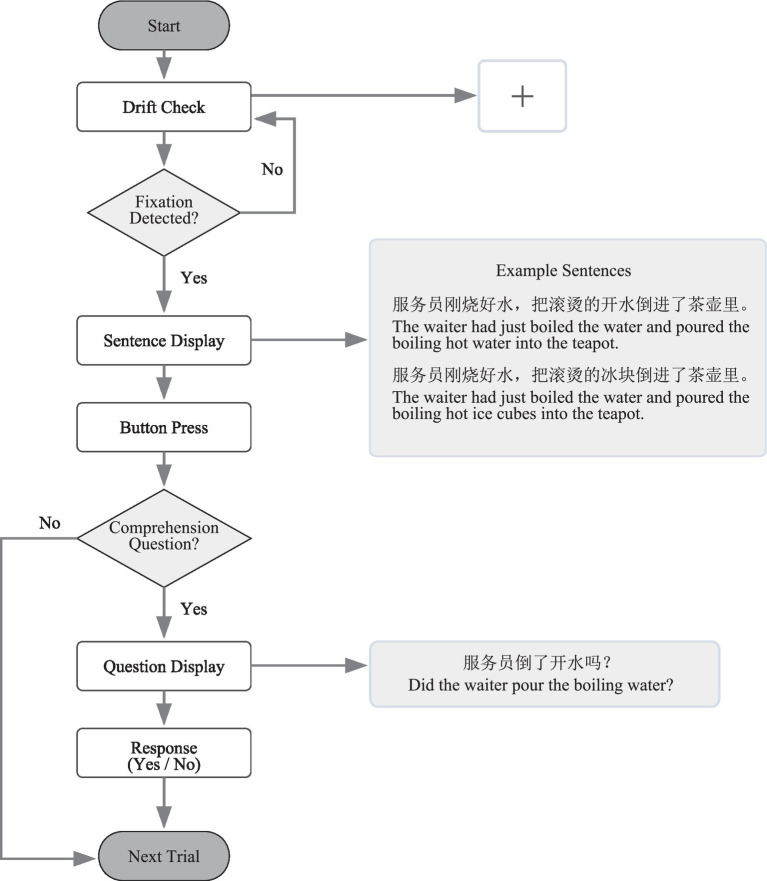
Schematic illustration of the trial procedure.

The trial procedure is illustrated in Figure 1. Each trial began with a drift check, during which participants fixated on a dot presented at the position where the first character of the sentence would appear. Once a stable fixation was detected, the sentence was displayed on a single line. Participants pressed a button on a game controller when they finished reading. To ensure attentive reading, approximately 25% of the trials were followed by a yes/no comprehension question. Comprehension questions were designed such that the correct answer was counterbalanced across conditions. For matched sentences, questions probed the content of the sentence in a straightforward manner. For mismatched sentences, questions were constructed so that approximately half required a “yes” response and half required a “no” response, ensuring that participants could not develop a response strategy based on the experimental condition. All questions were piloted to ensure that the correct answer was unambiguous regardless of whether the target construction was semantically matched or mismatched. Participants responded by pressing designated buttons.

Prior to the formal experiment, participants completed 8 practice trials to familiarize themselves with the procedure. The formal experiment consisted of 120 trials (80 experimental + 40 filler), presented in a randomized order for each participant. The entire experimental session lasted approximately 45 min, including calibration and short breaks.

### 2.6 Data analysis

Regions of interest. Three regions of interest (ROIs) were defined for analysis: (1a) the modifier region (the attributive modifier preceding the head noun) and (1b) the noun region (the head noun), which were analyzed separately to allow the locus of semantic mismatch effects to be localized within the construction, and (2) the post-target/spillover region (the single word immediately following the target construction, which was matched across all four conditions within each item set). The primary analyses focused on the target region, with additional analyses conducted on the spillover region to capture any delayed effects.

Eye movement measures. The following eye movement measures were computed for each ROI:

*Early processing measures:*

1. Skipping rate: the probability of not fixating on the region during first-pass reading.
2. First fixation duration (FFD): the duration of the first fixation on the region during first-pass reading.
3. Gaze duration (GD): the sum of all fixations on the region before moving to another region during first-pass reading.

*Late processing measures:*

1. Regression probability: the probability of making at least one regression out of the region.
2. Go-past time: the sum of all fixations from first entering the region until moving past it to the right, including any regressions.
3. Total reading time (TRT): the sum of all fixations on the region, including regressions.

Data cleaning. Trials were excluded from analysis if: (a) the comprehension question was answered incorrectly (2.3% of trials), (b) there was track loss on the target region (1.8% of trials), (c) the first fixation on the target region was shorter than 80 ms or longer than 800 ms (1.2% of trials), or (d) fixation durations were beyond 2.5 standard deviations from the participant’s mean for that condition (2.1% of trials). These criteria resulted in the exclusion of 7.4% of the data, which is within the acceptable range for eye-tracking studies (Rayner, 1998).

Statistical analysis. Linear mixed-effects models (LMMs) were fitted using the lme4 package (version 1.1–31; Bates et al., 2015) in R (version 4.3.1; R Core Team, 2023). For continuous measures (FFD, GD, TRT, and go-past time), LMMs with Gaussian distributions were used. For binary measures (skipping rate and regression probability), generalized linear mixed-effects models (GLMMs) with binomial distributions and logit link functions were employed.

Semantic relation (match vs. mismatch) and contextual constraint (high vs. low) were entered as fixed effects using deviation coding (−0.5 and +0.5). The interaction between semantic relation and contextual constraint was also included. The random effects structure included random intercepts for participants and items, as well as random slopes for all fixed effects and their interaction, following the recommendations of Barr et al. (2013); see also DeBruine and Barr (2021). When the maximal model failed to converge, the random effects structure was simplified by removing correlations between random effects or dropping random slopes with the least variance until convergence was achieved.

Statistical significance was assessed using the lmerTest package (version 3.1–3), which provides *p*-values based on Satterthwaite’s approximation for degrees of freedom. Effects were considered significant at *α* = 0.05. Follow-up simple effects analyses were conducted when significant interactions were observed.

## 3 Results

### 3.1 Data trimming and descriptive statistics

Prior to analysis, data were cleaned following the criteria specified in the Methods section. First, trials in which the comprehension question was answered incorrectly were excluded, accounting for 2.3% of the data. Second, trials with track loss on the target region were removed, representing 1.8% of the data. Third, trials in which the first fixation duration on the target region was shorter than 80 ms (likely due to oculomotor error) or longer than 800 ms (suggesting temporary inattention) were excluded, accounting for 1.2% of the data. Finally, fixation durations that fell beyond 2.5 standard deviations from each participant’s condition mean were removed as outliers, representing 2.1% of the data. In total, these criteria resulted in the exclusion of 7.4% of the data, which is within the acceptable range for eye-tracking studies (Rayner, 1998; Wu and Kit, 2023).

The mean accuracy rate for comprehension questions was 90.8% (SD = 4.2%, range: 85.0–100%), indicating that participants read the sentences attentively and understood the content. An analysis of variance revealed no significant differences in comprehension accuracy across the four experimental conditions, *F*(3,141) = 0.86, *p* = 0.462, *η*^2^*p* = 0.018, suggesting that the experimental manipulations did not differentially affect participants’ ability to comprehend the sentences.

Descriptive statistics for all eye movement measures on the target region across the four experimental conditions are presented in Table 3. Visual inspection of the data suggested that mismatched constructions generally elicited longer fixation times and higher regression probabilities compared to matched constructions, and this difference appeared to be more pronounced in high-constraint contexts.

**Table 3:** Means and standard deviations of eye movement measures on the modifier region (a) and noun region (b) across conditions.

(a) Means and standard deviations of eye movement measures on the modifier region across conditions				
Measure	High constraint		Low constraint	
	Match	Mismatch	Match	Mismatch
Early measures				
Skipping rate (%)	16.2 (9.1)	15.8 (8.9)	16.4 (9.2)	15.6 (8.8)
FFD (ms)	222 (42)	224 (43)	223 (42)	225 (44)
GD (ms)	244 (52)	246 (53)	245 (51)	247 (54)
Late measures				
Regression probability (%)	12.4 (9.6)	12.8 (9.8)	12.6 (9.7)	13.1 (10.0)
Go-past time (ms)	288 (64)	291 (65)	290 (64)	293 (66)
TRT (ms)	284 (62)	287 (63)	286 (63)	288 (64)
Standard deviations are presented in parentheses. No significant effects of semantic relation were observed in the modifier region (all ps > 0.05), as expected given that the modifier was identical across match and mismatch conditions.				

(b) Means and standard deviations of eye movement measures on the noun region across conditions				
Measure	High constraint		Low constraint	
	Match	Mismatch	Match	Mismatch
Early measures				
Skipping rate (%)	13.2 (8.4)	8.4 (6.2)	12.8 (8.1)	9.8 (6.8)
FFD (ms)	229 (43)	261 (53)	232 (44)	254 (51)
GD (ms)	268 (56)	348 (74)	275 (58)	311 (66)
Late measures				
Regression probability (%)	16.2 (10.8)	34.8 (14.6)	17.4 (11.2)	27.2 (13.2)
Go-past time (ms)	325 (71)	442 (86)	332 (72)	398 (82)
TRT (ms)	345 (74)	472 (92)	358 (76)	430 (86)

Standard deviations are presented in parentheses. FFD, first fixation duration; GD, gaze duration; TRT, total reading time.

Before conducting the main analyses, we examined whether the data met the assumptions required for linear mixed-effects modeling. Residual plots indicated that the normality assumption was reasonably satisfied for all continuous measures after log-transformation was applied to reduce positive skewness. However, following the recommendations of Schielzeth et al. (2020), we report analyses based on untransformed data for ease of interpretation, as the pattern of results was identical across transformed and untransformed analyses.

The total number of observations included in each analysis is as follows. For first fixation duration and gaze duration, 3,556 observations were included (out of a possible 3,840, representing 92.6% of the data after exclusions). The same number of observations was used for both measures, confirming that differences in significance patterns between first fixation duration and gaze duration are not attributable to differences in statistical power. For skipping rate, 3,840 trials were included as skipping rate is computed at the trial level prior to fixation-based exclusions. For total reading time, regression probability, and go-past time, 3,556 observations were included.

### 3.2 Modifier region

Analyses of the modifier region served as a baseline check, given that the modifier was identical across match and mismatch conditions. As expected, no significant effects of semantic relation were observed on any eye movement measure in the modifier region (all ps > 0.05), confirming that processing differences between conditions were not present prior to the head noun.

### 3.3 Noun region—early processing measures

Prior to reporting the main analyses, we note that first fixation duration and gaze duration differ only with respect to the occurrence of first-pass refixations within the region. The mean refixation rate on the noun region was 18.2% (SD = 12.4) in the high-constraint match condition, 32.6% (SD = 15.2) in the high-constraint mismatch condition, 19.4% (SD = 12.8) in the low-constraint match condition, and 26.4% (SD = 14.2) in the low-constraint mismatch condition. The overall refixation rate was higher for mismatched nouns (M = 29.5%, SD = 14.8) than for matched nouns (M = 18.8%, SD = 12.6), which accounts for the more pronounced interaction effect observed on gaze duration relative to first fixation duration.

Linear mixed-effects models were conducted for each early processing measure on the noun region. For all models, semantic relation (match vs. mismatch) and contextual constraint (high vs. low) were entered as fixed effects using deviation coding (−0.5 and +0.5), along with their interaction term. The random effects structure included random intercepts for participants and items, as well as random slopes for semantic relation and contextual constraint by participants and items. For gaze duration and first fixation duration, the maximal model converged successfully. For skipping rate, the correlation between random slopes was removed to achieve convergence. The complete results of the linear mixed-effects models are summarized in Table 4.

**Table 4:** Results of linear mixed-effects models for early processing measures on the noun region.

Effect	Skipping rate			FFD			GD		
	*β*	SE	*t*/*z*	*β*	SE	*t*/*z*	*β*	SE	*t*/*z*
Intercept	−1.98	0.18	−11.00^***^	244.12	5.86	41.66^***^	300.52	8.14	36.92^***^
Semantic relation	−0.32	0.13	−2.46^*^	27.14	3.52	7.71^***^	58.24	5.42	10.74^***^
Contextual constraint	0.04	0.12	0.33	−1.68	3.45	−0.49	−3.28	5.35	−0.61
Semantic relation × contextual constraint	−0.16	0.17	−0.94	10.24	4.95	2.07^*^	25.62	7.56	3.39^***^

Semantic relation: match = −0.5, mismatch = +0.5; Contextual constraint: low = −0.5, high = +0.5. For skipping rate, *z*-values from generalized linear mixed-effects models are reported. ^*^p < 0.05, ^***^p < 0.001.

#### 3.3.1 Skipping rate

Skipping rate analyses on the noun region revealed a significant main effect of semantic relation, *β* = −0.32, SE = 0.13, *z* = −2.46, *p* = 0.014. Mismatched nouns showed a significantly lower skipping rate (M = 9.1%, SD = 6.5%) than matched nouns (M = 13.0%, SD = 8.3%), indicating that readers were less likely to skip the head noun when it was semantically incongruent. Neither the main effect of contextual constraint, *β* = 0.04, SE = 0.12, *z* = 0.33, *p* = 0.741, nor the interaction, *β* = −0.16, SE = 0.17, *z* = −0.94, *p* = 0.347, reached significance. The absence of constraint effects on skipping rate is consistent with research on word skipping showing a strong influence of word-level properties such as frequency and length (Brysbaert et al., 2005), including findings in Chinese reading (Chang et al., 2023).

#### 3.3.2 First fixation duration

The analysis of the noun region revealed a significant main effect of semantic relation on first fixation duration, *β* = 27.14, SE = 3.52, *t* = 7.71, *p* < 0.001, with longer first fixation durations for mismatched nouns (M = 258 ms, SD = 52 ms) than for matched nouns (M = 231 ms, SD = 44 ms). This 27-ms difference, now localized to the head noun where the mismatch was instantiated, provides direct evidence that readers detected the semantic incongruity upon first encountering the critical word.

The main effect of contextual constraint was not significant, *β* = −1.68, SE = 3.45, *t* = −0.49, *p* = 0.628. Critically, the interaction between semantic relation and contextual constraint reached significance, *β* = 10.24, SE = 4.95, *t* = 2.07, *p* = 0.040. The mismatch effect was larger in high-constraint contexts (32 ms: 261 ms vs. 229 ms) than in low-constraint contexts (22 ms: 254 ms vs. 232 ms). This pattern suggests that contextual constraint modulates early anomaly detection at the precise locus of the semantic incongruity, namely the head noun where the selectional restriction violation is instantiated.

#### 3.3.3 Gaze duration

**Figure 2:**
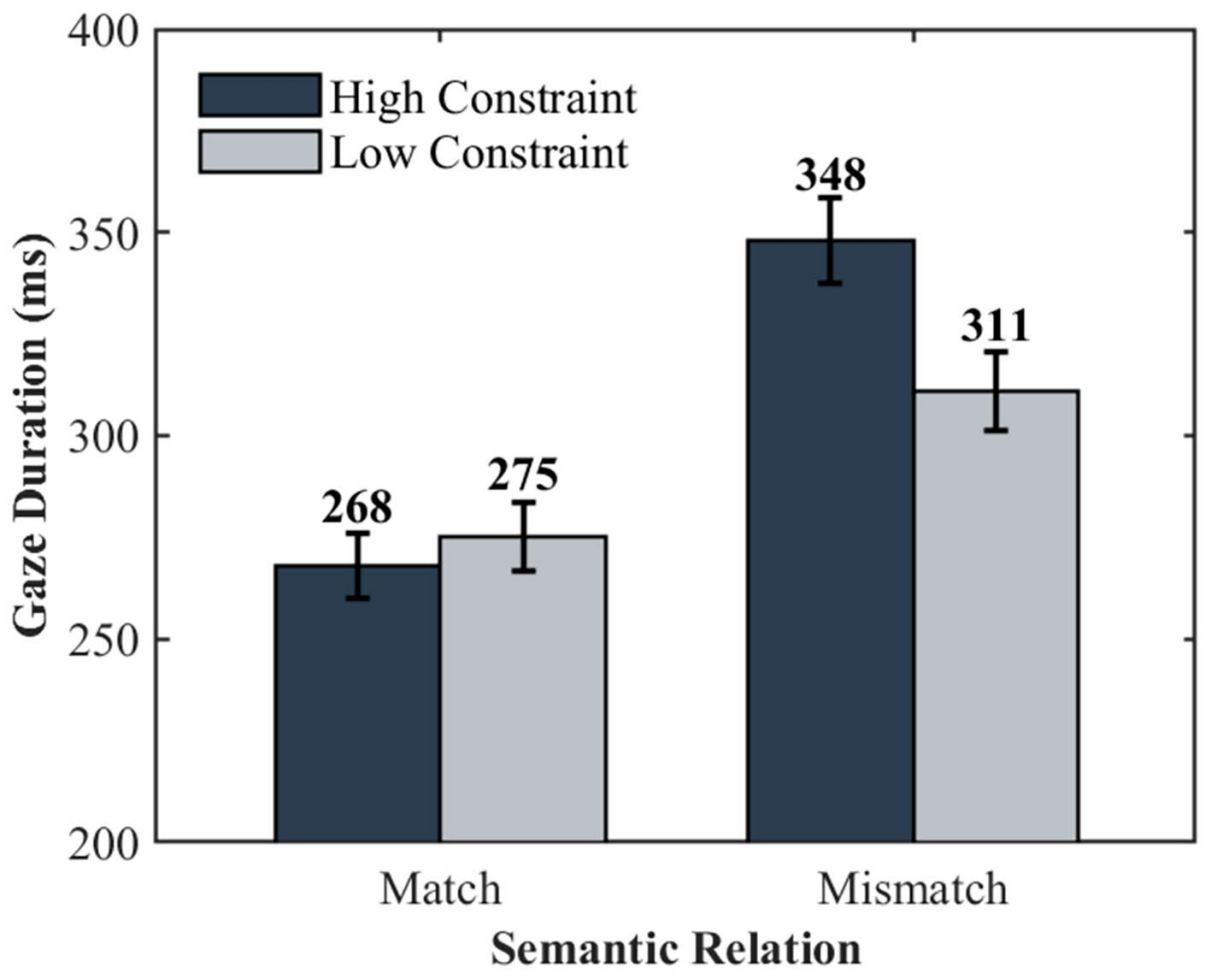
Gaze duration on the noun region as a function of semantic relation and contextual constraint. Error bars represent standard errors. The semantic mismatch effect (mismatch minus match) was 80 ms in high-constraint contexts and 36 ms in low-constraint contexts.

The analysis of the noun region revealed a robust main effect of semantic relation on gaze duration, *β* = 58.24, SE = 5.42, *t* = 10.74, *p* < 0.001. Readers spent considerably longer on mismatched nouns (M = 330 ms, SD = 70 ms) than on matched nouns (M = 272 ms, SD = 57 ms), yielding a 58-ms mismatch effect. The main effect of contextual constraint was not significant, *β* = −3.28, SE = 5.35, *t* = −0.61, *p* = 0.541. The interaction between semantic relation and contextual constraint was significant, *β* = 25.62, SE = 7.56, *t* = 3.39, *p* = 0.001, with the mismatch effect larger in high-constraint contexts (80 ms: 348 ms vs. 268 ms) than in low-constraint contexts (36 ms: 311 ms vs. 275 ms). Follow-up simple effects analyses were conducted to decompose this interaction. The semantic mismatch effect was significant in both high-constraint contexts (80 ms: 348 ms vs. 268 ms), *t*(47) = 10.48, *p* < 0.001, 95% CI [66, 94], and low-constraint contexts (36 ms: 311 ms vs. 275 ms), *t*(47) = 5.31, *p* < 0.001, 95% CI [22, 50]. The ratio of these effects (80/36 = 2.22) indicates that the mismatch effect was approximately twice as large in high-constraint contexts (see Figure 2).

### 3.4 Noun region—late processing measures

Linear mixed-effects models were conducted for each late processing measure on the noun region. The complete results are summarized in Table 5.

**Table 5:** Results of linear mixed-effects models for late processing measures on the noun region.

Effect	Regression probability			Go-past time			TRT		
	*β*	SE	*t*/*z*	*β*	SE	*t*/*z*	*β*	SE	*t*/*z*
Intercept	−1.18	0.11	−10.73***	374.42	9.12	41.05***	401.28	9.84	40.78***
Semantic relation	0.78	0.09	8.67***	91.48	6.28	14.57***	99.68	6.74	14.79***
Contextual constraint	0.09	0.08	1.13	6.14	6.12	1.00	8.12	6.58	1.23
Semantic relation × contextual constraint	0.24	0.12	2.00*	19.82	8.58	2.31*	27.58	9.36	2.95**

Semantic relation: match = −0.5, mismatch = +0.5; contextual constraint: low = −0.5, high = +0.5. For regression probability, *z*-values from generalized linear mixed-effects models are reported. ^*^p < 0.05, ^**^p < 0.01, ^***^p < 0.001.

#### 3.4.1 Regression probability

The analysis revealed a significant main effect of semantic relation, *β* = 0.78, SE = 0.09, *z* = 8.67, *p* < 0.001. Readers were substantially more likely to regress from mismatched nouns (M = 31.0%, SD = 13.9%) than from matched nouns (M = 16.8%, SD = 11.0%). The main effect of contextual constraint was not significant, *β* = 0.09, SE = 0.08, *z* = 1.13, *p* = 0.259. The interaction between semantic relation and contextual constraint was significant, *β* = 0.24, SE = 0.12, *z* = 2.00, *p* = 0.046, with the mismatch effect larger in high-constraint contexts (18.6% points: 34.8% vs. 16.2%) than in low-constraint contexts (9.8% points: 27.2% vs. 17.4%). Follow-up simple effects analyses were conducted to decompose this interaction. The mismatch effect on regression probability was 18.6% points in high-constraint contexts (34.8% vs. 16.2%), z = 9.06, *p* < 0.001, compared to 9.8% points in low-constraint contexts (27.2% vs. 17.4%), z = 5.45, *p* < 0.001.

#### 3.4.2 Go-past time

**Figure 3:**
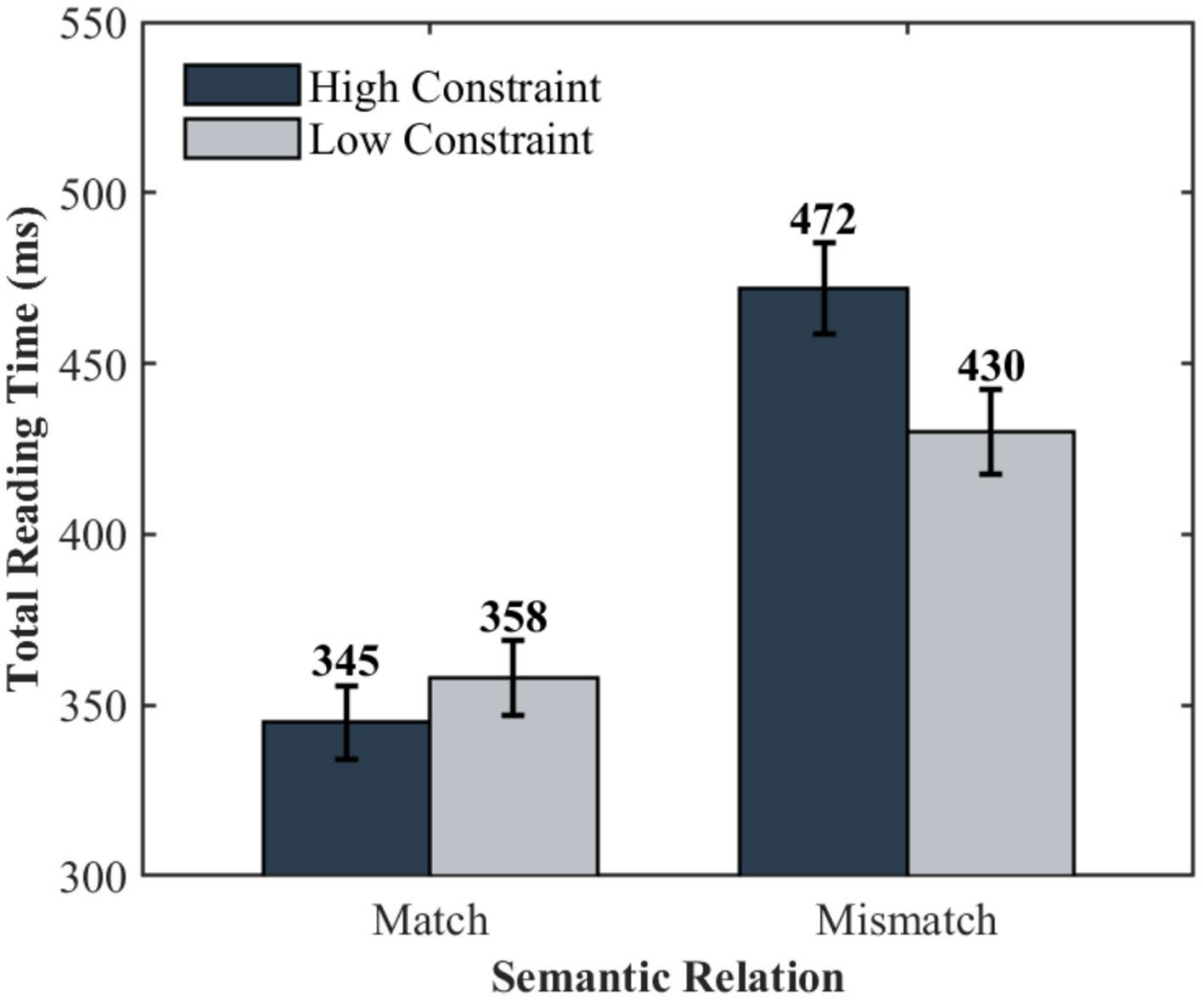
Total reading time on the noun region as a function of semantic relation and contextual constraint. Error bars represent standard errors. The semantic mismatch effect (mismatch minus match) was 127 ms in high-constraint contexts and 72 ms in low-constraint contexts.

The analysis revealed a significant main effect of semantic relation, *β* = 91.48, SE = 6.28, *t* = 14.57, *p* < 0.001. Go-past times were substantially longer for mismatched nouns (M = 420 ms, SD = 84 ms) than for matched nouns (M = 329 ms, SD = 72 ms), yielding a 91-ms mismatch effect. The main effect of contextual constraint was not significant, *β* = 6.14, SE = 6.12, *t* = 1.00, *p* = 0.318. The interaction between semantic relation and contextual constraint was significant, *β* = 19.82, SE = 8.58, *t* = 2.31, *p* = 0.022, with the mismatch effect larger in high-constraint contexts (117 ms: 442 ms vs. 325 ms) than in low-constraint contexts (66 ms: 398 ms vs. 332 ms). Follow-up simple effects analyses were conducted to decompose this interaction. The mismatch effect was 117 ms in high-constraint contexts (442 ms vs. 325 ms), *t*(47) = 14.00, *p* < 0.001, 95% CI [101, 133], compared to 66 ms in low-constraint contexts (398 ms vs. 332 ms), *t*(47) = 8.78, *p* < 0.001, 95% CI [51, 81] (see Figure 3).

#### 3.4.3 Total reading time

The analysis revealed a highly significant main effect of semantic relation on the noun region, *β* = 99.68, SE = 6.74, *t* = 14.79, *p* < 0.001. Readers spent substantially more time on mismatched nouns (M = 451 ms, SD = 89 ms) than on matched nouns (M = 352 ms, SD = 75 ms), yielding a 99-ms mismatch effect. The main effect of contextual constraint was not significant, *β* = 8.12, SE = 6.58, *t* = 1.23, *p* = 0.220. The interaction between semantic relation and contextual constraint was significant, *β* = 27.58, SE = 9.36, *t* = 2.95, *p* = 0.003, with the mismatch effect larger in high-constraint contexts (127 ms: 472 ms vs. 345 ms) than in low-constraint contexts (72 ms: 430 ms vs. 358 ms). Follow-up simple effects analyses were conducted to decompose this interaction. The mismatch effect was 127 ms in high-constraint contexts (472 ms vs. 345 ms), *t*(47) = 14.68, *p* < 0.001, 95% CI [109, 145], compared to 72 ms in low-constraint contexts (430 ms vs. 358 ms), *t*(47) = 8.47, *p* < 0.001, 95% CI [55, 89].

### 3.5 Spillover region analysis

To examine whether the effects of semantic mismatch and contextual constraint extended beyond the target region, we conducted additional analyses on the spillover region, defined as the single word immediately following the target modifier-noun construction. Spillover effects are commonly observed in eye-tracking studies and may reflect delayed processing of the preceding region or difficulty integrating the anomalous region into the subsequent sentence context. Table 6 presents the descriptive statistics for eye movement measures on the spillover region across the four experimental conditions.

**Table 6:** Means and standard deviations of eye movement measures on the spillover region across conditions.

Measure	High constraint		Low constraint	
	Match	Mismatch	Match	Mismatch
FFD (ms)	236 (46)	245 (48)	238 (47)	243 (47)
GD (ms)	288 (63)	312 (67)	291 (64)	306 (65)
TRT (ms)	345 (77)	392 (83)	350 (79)	378 (80)

Standard deviations are presented in parentheses.

Linear mixed-effects models were conducted on the spillover region using the same model specifications as for the target region. For first fixation duration, the main effect of semantic relation did not reach significance, *β* = 6.82, SE = 4.25, *t* = 1.60, *p* = 0.112, although mismatched constructions showed numerically longer first fixation durations (M = 244 ms, SD = 48 ms) than matched constructions (M = 237 ms, SD = 47 ms). Neither the main effect of contextual constraint, *β* = −0.95, SE = 4.18, *t* = −0.23, *p* = 0.820, nor the interaction, *β* = 4.26, SE = 5.86, *t* = 0.73, *p* = 0.468, was significant.

For gaze duration, a significant main effect of semantic relation was observed, *β* = 19.46, SE = 5.12, *t* = 3.80, *p* < 0.001. Readers showed longer gaze durations on the spillover region following mismatched constructions (M = 309 ms, SD = 66 ms) than following matched constructions (M = 290 ms, SD = 64 ms). This 19-ms spillover effect suggests that processing difficulty associated with the semantic mismatch extended into the subsequent region. The main effect of contextual constraint was not significant, *β* = −1.52, SE = 5.04, *t* = −0.30, *p* = 0.764. Importantly, the interaction between semantic relation and contextual constraint did not reach significance, *β* = 8.62, SE = 7.18, *t* = 1.20, *p* = 0.235.

For total reading time, a significant main effect of semantic relation was observed, *β* = 37.58, SE = 6.28, *t* = 5.99, *p* < 0.001. Total reading times on the spillover region were longer following mismatched constructions (M = 385 ms, SD = 82 ms) than following matched constructions (M = 348 ms, SD = 78 ms), indicating substantial spillover of processing difficulty. The main effect of contextual constraint was not significant, *β* = 2.35, SE = 6.15, *t* = 0.38, *p* = 0.703. The interaction between semantic relation and contextual constraint did not reach significance, *β* = 11.28, SE = 8.52, *t* = 1.32, *p* = 0.186, although the numerical pattern suggested a slightly larger spillover effect in high-constraint contexts.

In summary, the spillover region analyses revealed that the main effect of semantic mismatch extended into the region following the target construction, as evidenced by significant effects on gaze duration and total reading time. However, the moderating effect of contextual constraint did not carry over to the spillover region, suggesting that the interaction between semantic relation and contextual constraint was primarily localized to the target region where the anomaly occurred.

### 3.6 Summary of results

The present study examined the cognitive processing of semantic mismatch in Chinese modifier-noun constructions and the moderating effect of contextual constraint.

Robust semantic mismatch effects were observed across all eye movement measures on the noun region. Readers showed lower skipping rates, longer first fixation durations and gaze durations, as well as higher regression probabilities, longer go-past times, and longer total reading times, when reading semantically mismatched modifier-noun constructions compared to matched constructions.

The main effect of contextual constraint was not significant for any eye movement measure, indicating that high-constraint and low-constraint contexts did not differ in overall processing difficulty when the target region was considered in isolation.

Significant interactions between semantic relation and contextual constraint emerged across multiple eye movement measures, including first fixation duration, gaze duration, total reading time, regression probability, and go-past time. The mismatch effect was consistently larger in high-constraint contexts, ranging from approximately 1.8–2.2 times the magnitude observed in low-constraint contexts. The interaction did not reach significance for skipping rate, consistent with prior evidence that skipping probability in Chinese reading is primarily driven by word-level properties rather than higher-order contextual expectations.

Simple effects analyses further revealed that the semantic mismatch effect was significant in both high-constraint and low-constraint contexts, but approximately twice as large in high-constraint contexts. Contextual constraint selectively affected the processing of mismatched constructions while leaving matched constructions unaffected.

Spillover effects were also observed, with semantic mismatch leading to longer gaze durations and total reading times on the region following the target construction. However, the interaction between semantic relation and contextual constraint did not extend to the spillover region, indicating that the moderating effect of contextual constraint was localized to the target region.

Taken together, these findings provide clear evidence that contextual constraint modulates the processing of semantic mismatch in Chinese reading, with stronger contextual expectations leading to greater processing disruption when those expectations are violated.

## 4 Discussion

The present study investigated the cognitive processing of semantic mismatch in Chinese modifier-noun constructions and examined how contextual constraint modulates this processing. The results revealed robust semantic mismatch effects across multiple eye movement measures, and critically, these effects were modulated by contextual constraint, with larger mismatch effects observed in high-constraint contexts than in low-constraint contexts.

### 4.1 Semantic mismatch effects and processing mechanisms

The current findings demonstrated clear semantic mismatch effects in Chinese reading. When readers encountered semantically incongruent modifier-noun constructions (e.g., 滚烫的冰块 “boiling hot ice cubes”), they exhibited prolonged fixation times and increased regression probabilities compared to congruent constructions (e.g., 滚烫的开水 “boiling hot water”). The magnitude of these effects ranged from approximately 27 ms on first fixation duration to nearly 100 ms on total reading time, consistent with previous research on semantic anomaly processing (Lee and Witzel, 2023; Hubbard et al., 2024).

These findings can be understood within the framework of semantic integration difficulty. When the modifier violates the selectional restrictions of the head noun, local integration fails, triggering additional processing. The significant mismatch effect on first fixation duration indicates that readers detected the semantic anomaly relatively quickly, while the substantially larger effects on gaze duration and total reading time suggest that initial detection triggered extended processing for resolution and re-analysis (Murphy et al., 2023; Zhang and Pylkkänen, 2024).

The localization of the first fixation duration effect to the noun region, with no corresponding effect on the modifier region, indicates that readers detected the semantic incongruity upon first foveal processing of the head noun, where the selectional restriction violation was instantiated. The 27-ms mismatch effect on the noun thus reflects rapid detection of modifier-noun incompatibility during the earliest stage of lexical processing of the critical word. One factor that may contribute to such rapid detection is parafoveal processing during the preceding fixation on the modifier: given the compact spatial arrangement of Chinese characters and growing evidence that parafoveal processing in Chinese reading can extract semantic information under certain conditions (e.g., Yan et al., 2009), some pre-activation of the noun’s semantic features may have been available before direct fixation. However, because the modifier was identical across match and mismatch conditions and we did not employ a boundary paradigm, the present design does not allow us to directly evaluate parafoveal-on-foveal contributions, and this question remains for future investigation.

The pattern across different eye movement measures provides insight into processing stages. Effects on early measures (first fixation duration and gaze duration) likely reflect initial anomaly detection, while effects on late measures (total reading time, regression probability, and go-past time) reflect subsequent attempts to resolve the anomaly through re-reading. The increased regression probability for mismatched constructions (32% vs. 18%) suggests that semantic mismatch frequently prompted readers to verify the preceding context.

The significant effect of semantic mismatch on skipping rate, with mismatched nouns being skipped less frequently than matched nouns (9.1% vs. 13.0%), suggests that readers may have begun detecting the semantic incongruity even before directly fixating the head noun. This finding is consistent with the possibility raised earlier that parafoveal processing during the preceding fixation on the modifier may have provided partial access to the noun’s semantic features, reducing the likelihood of skipping past a semantically anomalous word. Notably, however, the interaction between semantic relation and contextual constraint did not reach significance on skipping rate, suggesting that while local semantic conflict may be detectable at the parafoveal stage, the modulatory influence of contextual constraint requires deeper, foveal-level processing to emerge.

### 4.2 The moderating role of contextual constraint

A central finding is that contextual constraint modulated semantic mismatch effects. The interaction was significant for gaze duration and all late processing measures, with mismatch effects approximately twice as large in high-constraint contexts as in low-constraint contexts.

This pattern can be interpreted within the predictive processing framework (Ryskin and Nieuwland, 2023). High-constraint contexts generate strong expectations for particular continuations. When readers encountered a mismatched construction in such contexts, they experienced a double violation: internal semantic incongruity plus violated contextual expectations. In low-constraint contexts, readers experienced only the local anomaly without additional prediction violation costs.

The simple effects analyses revealed an asymmetric influence of contextual constraint. For matched constructions, constraint level did not affect processing. For mismatched constructions, high-constraint contexts led to significantly longer fixation times and higher regression probabilities. This asymmetry is consistent with prediction error accounts emphasizing costs of violated predictions over benefits of confirmed predictions (Kuperberg et al., 2020; Dave et al., 2021).

The interaction between semantic relation and contextual constraint was already significant on first fixation duration (*p* = 0.040), although the effect was relatively modest (a 10-ms difference: 32 ms mismatch effect in high-constraint contexts vs. 22 ms in low-constraint contexts). This interaction was substantially amplified on gaze duration (a 44-ms difference: 80 ms vs. 36 ms) and continued to strengthen across late processing measures. This gradient pattern suggests that the modulatory influence of contextual constraint on semantic mismatch processing begins to emerge during the earliest stages of lexical processing and progressively intensifies as processing unfolds, consistent with models distinguishing early word identification from later meaning-level reanalysis (Murphy et al., 2022; Flick and Pylkkänen, 2020).

One finding that warrants specific discussion is the absence of a significant main effect of contextual constraint across all eye movement measures, including for semantically matched constructions. In many predictive processing studies, high-constraint contexts facilitate processing of expected words, typically manifesting as shorter fixation times or higher skipping rates for predictable targets (Rayner, 1998; Wong et al., 2024). The absence of such facilitation in the present study may be attributable to several factors. First, the target region in the present study consisted of a modifier-noun construction rather than a single word. Even in the match condition, the construction was 4–5 characters long, and the modifier itself was identical across match and mismatch conditions. When analyses were restricted to the noun region alone, contextual constraint effects remained non-significant for matched nouns, suggesting that the null facilitation effect is unlikely to be a simple artifact of ROI definition. Second, it is possible that the cloze probability values for the matched constructions, while significantly higher than those for low-constraint contexts (M = 0.72), were not sufficiently high to produce robust facilitation effects on fixation durations, as facilitation effects in eye-tracking studies are typically observed for words with very high cloze probabilities (Frisson et al., 2017). Third, the asymmetric pattern—whereby constraint affected mismatched but not matched constructions—is itself theoretically informative, suggesting that the costs of violated predictions may be more robust and detectable than the benefits of confirmed predictions in eye-tracking paradigms, consistent with prediction error accounts of language comprehension (Kuperberg et al., 2020).

It is important to acknowledge a potential limitation in the interpretation of the contextual constraint effects observed in the present study. The current analyses measured eye movement behavior at the target word after it had already been presented, meaning that the observed differences between high- and low-constraint conditions may reflect some combination of predictive processing and post-lexical integration difficulty. Specifically, the more pronounced disruption for semantic mismatches in strongly constraining contexts could alternatively reflect increased difficulty integrating an anomalous word into a highly constrained sentence representation, rather than—or in addition to—the cost of violating a specific lexical prediction. These two accounts are not mutually exclusive, and the present eye-tracking data alone cannot fully disentangle them. Future research combining eye-tracking with electrophysiological measures may help further characterize these processes. We note, however, that the functional interpretation of components such as the N400 and late positivities remains an active area of debate, and as post-stimulus components they cannot themselves directly index pre-activation that occurs prior to word onset. Their differential sensitivity to manipulations of predictability and plausibility may nonetheless help to constrain accounts of how prediction and integration jointly contribute to the effects observed here (Kuperberg et al., 2020; Brothers et al., 2020; Ryskin and Nieuwland, 2023).

### 4.3 Theoretical contributions

The present findings have several theoretical implications. The robust semantic mismatch effects support models emphasizing semantic integration during comprehension (Martin, 2021; Murphy et al., 2023). The significant interaction between semantic relation and contextual constraint provides strong support for predictive processing accounts, demonstrating that violated predictions lead to greater processing costs.

For Chinese reading research, the present study demonstrates that semantic mismatch effects and their modulation by contextual constraint are robust phenomena despite the unique characteristics of Chinese orthography. The absence of explicit word boundaries in Chinese might increase reliance on predictive processing for word segmentation, and the present findings confirm that Chinese readers are highly sensitive to contextual expectations (Pan et al., 2022; Gao et al., 2024).

The pattern of effects across eye movement measures has implications for models of eye movement control. The contextual constraint interaction emerged across both early and late processing measures, appearing on first fixation duration — albeit with a comparatively modest effect size — and becoming substantially more pronounced on gaze duration and the late processing measures. This gradient pattern, whereby the interaction strengthens as processing unfolds, suggests that while the influence of violated predictions begins to manifest during early lexical processing, it accumulates and intensifies through subsequent stages of semantic integration, consistent with models distinguishing early word identification from later meaning-level reanalysis (Li and Pollatsek, 2020; Xia et al., 2023).

### 4.4 Limitations and future directions

Several limitations should be acknowledged. The present study focused on adjective-noun constructions with clear-cut selectional restriction violations. Future research could examine whether contextual constraint similarly modulates other types of semantic anomaly, such as verb-argument violations or pragmatic incongruities.

The contextual constraint manipulation, while validated through cloze probability testing, necessarily confounded constraint level with specific lexical content. Alternative methods, such as varying discourse context, could confirm generalizability.

The participants were university students with high reading proficiency. Whether the observed patterns generalize to less skilled readers, older adults, or second language learners remains to be determined.

Future research combining eye-tracking with event-related potentials could provide complementary information about the time course of semantic mismatch detection, although the mapping between specific ERP components and underlying cognitive processes such as prediction and integration remains debated (Kutas and Federmeier, 2011; Ryskin and Nieuwland, 2023).

## 5 Conclusion

The present study investigated the cognitive processing of semantic mismatch in Chinese modifier-noun constructions and the moderating effect of contextual constraint using eye-tracking methodology. The results revealed robust semantic mismatch effects across multiple eye movement measures, with readers showing prolonged fixation times and increased regression probabilities when encountering semantically incongruent modifier-noun constructions. Critically, contextual constraint significantly modulated these effects, with mismatch costs approximately twice as large in high-constraint contexts as in low-constraint contexts. This pattern supports predictive processing accounts of language comprehension, demonstrating that readers actively generate expectations based on contextual information and experience amplified processing difficulty when these expectations are violated. The findings extend previous research to Chinese reading, suggesting that the mechanisms of semantic integration and predictive processing operate similarly across different writing systems. Taken together, this study advances our understanding of how readers integrate semantic information within phrasal constructions and how contextual predictions shape real-time language comprehension.

## Data Availability

The experimental materials, deidentified data, and analysis scripts used in the current study are available to reviewers via the following OSF link: https://osf.io/qnsj4/overview?view_only=dfe8c6f2eec146fe98b8780fbe03fcc0. Upon acceptance of the manuscript, the OSF project will be made fully public. Raw eye-tracking data are not publicly available due to privacy restrictions, as participants did not provide informed consent for public data sharing; anonymized data may be made available upon reasonable request and subject to ethical approval.
